# Evaluation of computer-aided detection for gastric cancer using white-light and linked-color imaging: a pilot study

**DOI:** 10.1016/j.igie.2025.09.010

**Published:** 2025-09-23

**Authors:** Takeshi Yasuda, Narutoshi Ando, Tamae Hashimoto, Yoshiaki Kanai, Yoichi Sakamoto, Yuki Endo, Tomohiro Soda, Takako Akazawa, Tsuguhiro Matsumoto, Norihito Yamauchi, Akira Muramatsu, Hiromu Kutsumi

**Affiliations:** Department Gastroenterology, Akashi City Hospital, Hyogo, Japan

## Abstract

**Background and Aims:**

In recent years, the field of endoscopic artificial intelligence has seen significant advancements, largely because of the widespread implementation of deep learning techniques. However, the computer-aided detection (CADe) of the stomach poses significant challenges in clinical practice. Here, we evaluated the performance of a newly developed CADe system, CAD EYE (Fujifilm, Tokyo, Japan), by comparing the frequency of the detection box appearance with white-light imaging (WLI) versus linked-color imaging (LCI) during the process of detecting gastric cancer (GC) and detection of GC with and without CADe.

**Methods:**

This single-center observational retrospective study included 105 patients who underwent esophagogastroduodenoscopy (EGD) using CADe and 105 controls selected by propensity-score matching from 600 patients. The primary outcome was to compare the detection box appearance of WLI and LCI during the CADe observation. Secondary outcomes included comparisons of biopsy rates, examination times, and cancer detection rates between groups. Furthermore, we investigated whether the landmark checker could accurately identify the stomach site.

**Results:**

CADe exhibited an average of 6.2 false-positive detections per case. False-positive rates were significantly lower with LCI than with WLI (3.48 vs 7.70, *P* < .001). The GC detection rate was higher in the CADe group than in the control group (4.8% vs 1.8%, *P* = .07), although the difference was not statistically significant. Biopsy rates and examination times were comparable between the groups. CADe accurately detected all 18 known-early GC cases. The landmark checker function identified an average of 5.72 of 7 key gastric sites (81.7%).

**Conclusions:**

This pilot study suggests that CADe, particularly when combined with LCI, may enhance GC detection during EGD without significantly increasing the examination time. Although promising, a high false-positive rate indicates that further optimization is needed.

## Introduction

Esophagogastroduodenoscopy (EGD) is widely used to detect early gastrointestinal (GI) tumors. However, in a multicenter retrospective cohort study, 8.7% of gastric cancers (GCs) were detected by EGD within 1 year after endoscopic submucosal dissection.^1^ Another multicenter retrospective cohort study reported that 8.9% of all GCs detected by EGD were submucosal invasive cancers, even if the patient had undergone EGD within 1 year of the previous procedure.^2^ Many of these GCs were likely to be missed lesions. The same is true for colonoscopy, where approximately 22% of small polyps are reported to be missed during a single endoscopic observation.^3^ Computer-aided detection (CADe) and computer-aided diagnosis (CADx) technologies have been developed using artificial intelligence (AI) technologies to reduce this rate. In particular, research on endoscopic AI has progressed rapidly over the past several years with the widespread adoption of deep learning technology.^4^, ^5^, ^6^ In the field of colonoscopy, EndoBRAIN-EYE (Olympus, Cybernet Systems, Tokyo, Japan),^7^ developed by Mori et al,^7^ and CAD EYE (CE) (Fujifilm, Tokyo, Japan)^8^ are routinely used in clinical practice in Japan. In 2024, CADe for colonoscopic assistance became an insured treatment in Japan. The development of AI for detecting and diagnosing early GC has progressed across various fields, demonstrating high precision and sensitivity in the research phase.^6^^,^^9^, ^10^, ^11^, ^12^ Additionally, AI for determining the imaging site of the upper GI tract has been developed and recently launched.^13^^,^^14^ However, unlike colonoscopy, the stomach—frequently affected by chronic inflammation from *Helicobacter pylori (H pylori)* infection in the background mucosa—continues to pose significant challenges for CADe in clinical practice. Currently, only 2 AI systems for the upper GI tract are available for clinical use in Japan: CE as CADe and gastroAI-model G (AI Medical Service, Tokyo, Japan) as CADx. Although CE has been available for more than 2 years since 2022, no validation data from actual clinical practice have been reported.

This study aimed to evaluate the performance of CADe for GC detection during EGD, with separate analyses for white-light imaging (WLI) and linked-color imaging (LCI), and to investigate the false-positive rate of CADe. Additionally, propensity-score matching was performed to retrospectively compare the GC detection rates between EGD procedures with and without CADe.

## Methods

### Study design

This study was conducted as a pilot study to evaluate the feasibility and potential utility of the CADe system. Data were collected retrospectively at a single center. No formal power calculation was performed because of the exploratory nature of the study.

At our institution, all endoscopic procedures are routinely recorded in video format. For this study, we reviewed all stored videos of CADe-assisted procedures conducted during the study period. The number of CADe detection boxes displayed during each examination was manually counted through careful frame-by-frame review of each video. This retrospective video review enabled accurate documentation of CADe activation, despite the absence of real-time manual annotation during the original procedures.

### Primary and secondary outcomes

The primary outcome of this study was to compare the false-positive detection rates of the CADe system between WLI and LCI. False-positive detections were defined as CADe-generated detection boxes that did not correspond to clinically diagnosed GCs.

The secondary outcomes included (1) characterization of false-positive detection boxes regarding location, surface color, and morphologic appearance; (2) comparison of biopsy rates, total examination time, and GC detection rates between CADe-assisted and conventional EGD performed during the same period; (3) evaluation of the landmark checker's stomach site recognition accuracy; and (4) assessment of whether CADe successfully detected all known upper GI cancers that were endoscopically visualized during the study period.

### Data collection

In this study, we included EGD examinations performed for a general assessment. These comprised endoscopic examinations for patients with mild abdominal symptoms, *H pylori* current infection or posteradication status, and follow-up after endoscopic treatment for early GCs. Patients who underwent EGD for therapeutic procedures such as hemostasis or for specialized examinations such as endoscopic ultrasonography were excluded.

Consecutive patients who underwent EGD with CADe were analyzed for this study between November 2023 and March 2024. During the same period, all patients who underwent EGD without CADe were retrospectively reviewed and used as controls. Our endoscopy unit operates several endoscopic systems, with CADe installed on 1 unit. Patients were randomly assigned to the endoscopic systems by the scheduling nurse team, and all examinations conducted with the CADe-equipped system used the CADe function. The control group served as a reference for secondary outcomes to assess whether the implementation of CADe influenced clinical workflow parameters (eg, biopsy rate, procedure time) and overall cancer detection rates.

### Reference standard for lesion detection

The reference standard for lesion detection was established as follows: when CADe indicated a lesion, the performing endoscopist evaluated whether the region appeared suspicious for neoplasia. If deemed suspicious, a biopsy was performed, and the histopathologic diagnosis served as the reference standard. For regions not considered suspicious by the endoscopist and therefore not biopsied, all detection boxes were retrospectively reviewed by a board-certified endoscopist using the recorded videos. This double-check process aims to confirm the appropriateness of the initial judgment. No additional suspicious lesions were identified during the secondary review.

### Equipment

A BL-7000 (Fujifilm) was used as the light-emitting diode (LED) light source. The VP-7000 (Fujifilm) served as the endoscopic system processor. The EG-760Z (Fujifilm) was used as the LED endoscope. The LED endoscope settings were configured as follows: WLI, H/+4/+4; LCI, B8/C3.

### Endoscopists

Board-certified fellows were endoscopic specialists or trainers certified by the Japan Gastroenterological Endoscopy Society. In total, 11 endoscopists conducted endoscopic examinations, 9 of whom were board-certified fellows, and the remaining 2 were noncertified endoscopists. The board-certified endoscopists had between 10 and 25 years of clinical experience as endoscopists, whereas both noncertified endoscopists were in their fourth year of clinical training. As CADe had only recently been introduced at our institution, none of the endoscopists—regardless of their certification status—had received specific training or possessed prior experience with the system.

### Computer-aided detection

**Figure 1:**
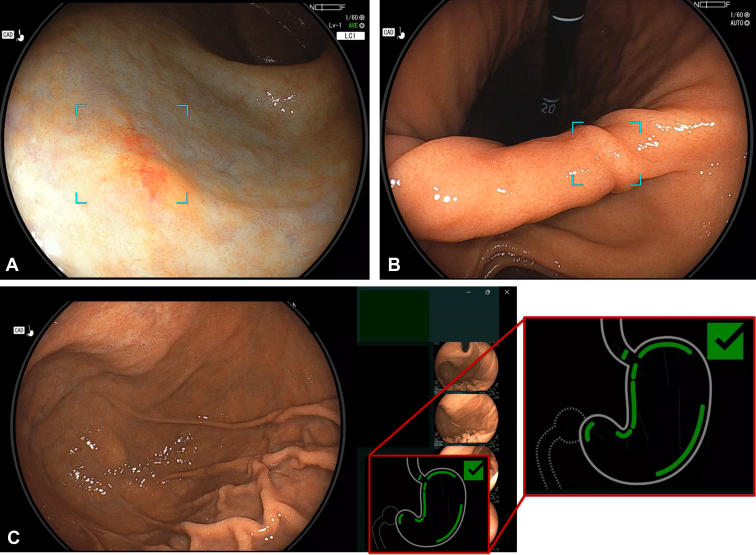
CAD EYE (Fujifilm, Tokyo, Japan) in clinical practice. **A,** Gastric cancer detected using CADe (inside *blue box*). **B,** Example of false-positive area indicated by CADe (inside *blue box*, normal mucosa). **C,** Landmark checker of CADe that recognizes 7 areas of the stomach wall (*green lines* in the cartoon of the stomach). The *check mark* indicates all areas have been examined. *CADe*, Computer-aided detection.

As a CADe for the identification of upper GI lesions, the CE system (EW10-EG01, version 1.1; Fujifilm) was used. When a lesion is detected, it is highlighted with a light-blue annotation box by CADe under both WLI and LCI modes (Fig. 1A).^8^ However, because the system prioritizes the prevention of oversight, it occasionally detects areas that are not tumorous (Fig. 1B).

CE uses a proprietary deep learning–based algorithm for real-time detection of gastric lesions. Although the detailed architecture and training data set characteristics remain proprietary because of commercial restrictions, prior studies have reported that the system uses convolutional neural networks trained on annotated endoscopic image data sets. Version 1.1, used in this study, features improved sensitivity and reduced detection box display latency compared with previous versions, based on manufacturer-reported performance updates.^9^ Here, patients who used CADe were observed by both WLI and LCI in their stomachs. The CE system also incorporates a “landmark checker” function that automatically detects and labels 7 major anatomical regions of the stomach, including the esophagogastric junction, antrum, angle/middle body/upper body of the lesser curvature, cardia, and body of the greater curvature, during the examination (Fig. 1C). This assists endoscopists in ensuring that all key gastric areas are visualized during the procedure.

### Quality assessment of diagnostic accuracy studies in artificial intelligence

In accordance with the QUAIDE (Quality Assessment of preclinical AI studies in Diagnostic Endoscopy) criteria,^15^ we evaluated our study design and reporting quality. This study satisfied several essential aspects: use of consecutive real-world cases, comparison with conventional endoscopic procedures (control group), inclusion of external clinical settings, assessment of key diagnostic performance metrics (eg, sensitivity, false-positive rate, area under the curve), and clear documentation of the AI tool and its integration into the clinical workflow. These elements align with the core domains of the QUAIDE framework and support the methodologic validity of our study.

### Definitions

False-positive detection boxes were defined as CADe-generated boxes that did not correspond to pathologically confirmed GC or lesions considered suspicious by the endoscopist.

*H pylori* infection status was evaluated using at least one of the following tests: a serum antibody test (baseline level: 0-10.0 U/mL), stool antigen test (positive or negative), Carbon-13-urea breath test (baseline level: 0%-2.4%), or histologic examination. The atrophic border of the background gastric mucosa was classified based on the Kimura-Takemoto classification.^16^ Regarding the observation time for EGD, the entire time from insertion to removal of the endoscope was calculated, including the time for magnification, dye spreading, and biopsy.

### Statistical analysis

Quantitative data were expressed as mean (standard deviation). Statistical significance was set at *P* <.05. Comparisons between the observed frequencies were performed using Fisher exact test, and nonparametric statistical analyses were performed using the Wilcoxon rank sum test. We performed propensity-score matching to control the effects of selection bias between the CADe and control groups.^17^ Age, sex, and history of GC were selected as matching variables because of their potential association with the selection process. Propensity scores were calculated using a logistic regression analysis. A propensity score–matched cohort was established by pairing each individual in the CADe group with a corresponding individual in the control group (1:1 matching), using the greedy matching algorithm. Subsequently, the matched cohorts were analyzed and compared. All statistical analyses were performed using JMP Pro version 17.00 (SAS International Inc, Cary, NC, USA), EZR version 1.63 (Saitama Medical Center, Jichi Medical University, Saitama City, Japan), and GraphPad Prism 9.00 for Windows (GraphPad Software, La Jolla, Calif, USA).

## Results

### Characteristics of patients and background data

**Figure 2:**
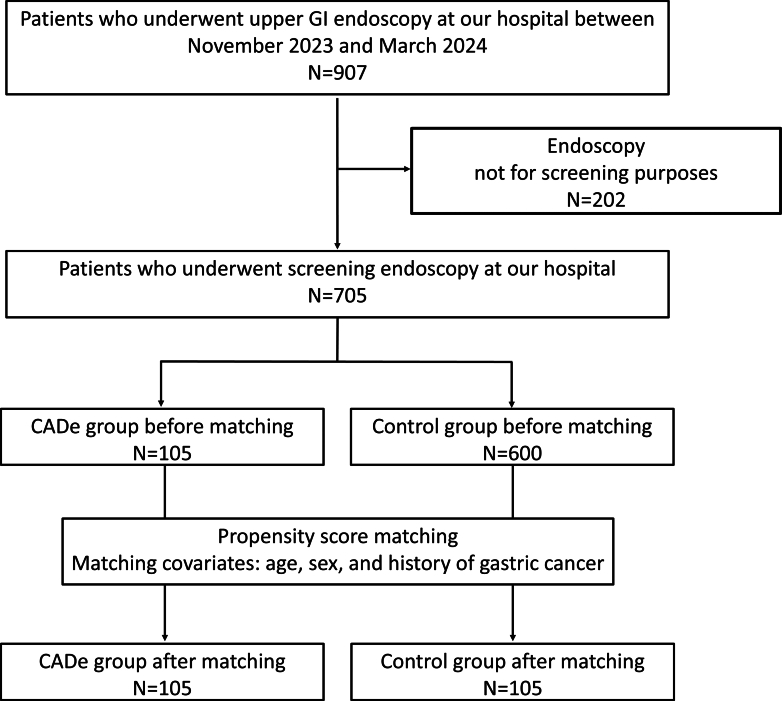
Flowchart of the patients recruited in this study. *CADe*, Computer-aided detection; *GI*, gastrointestinal.

From November 2023 to March 2024, a total of 907 patients underwent EGD at our hospital, and 202 were excluded from this study because they underwent therapeutic endoscopy or detailed evaluation (Fig. 2). Among the remaining 705 patients, 105 underwent EGD with CADe (CADe group) and 600 underwent EGD without CADe (control group). No patients opted out. The patient backgrounds are shown in Table 1. Patients were matched based on age, sex, history of GC, atrophic border, *H pylori* infection, and endoscopist level as covariates, with 105 patients selected from each group using propensity-score matching. The area under the receiver operating characteristic curve for the propensity score model was 0.612 (95% confidence interval [CI], 0.555-0.670), indicating moderate discriminative ability.

**Table 1:** Patient characteristics

Value	CADe group (n=105)	Control group before matching (n=600)	*P* value	Control group after matching (n=105)	*P* value	SMD
Age (year, mean (SD))	68.9 (13.5)	70.2 (11.7)	.54	67.7 (11.6)	.33	0.10
Sex (n (%))					.89	0.02
men	52 (49.5)	329 (54.8)	.34	53 (50.5)		
women	53 (50.5)	271 (45.2)		52 (49.5)		
History of GC (n (%))	12 (11.4)	64 (10.7)	.86	15 (14.3)	.68	0.09
Atrophic border∗ (n (%))					.87	0.02
Closed type	80 (76.2)	416 (69.3)	.16	81 (77.1)		
Open type	25 (23.8)	184 (30.7)		24 (22.9)		
*H.pylori* infection (n (%))					.51	0.16
Current infection	16 (15.2)	138 (23.0)	.09	22 (21.0)		
Past infection	50 (47.6)	227 (37.8)		44 (41.9)		
Uninfection	39 (37.1)	235 (39.2)		39 (37.1)		
Endoscopists (n (%))					.86	0.02
Board certified fellow	84 (80.0)	440 (73.3)	.18	85 (81.0)		
Young resident	21 (20.0)	160 (26.7)		20 (19.1)		

*CADe*, Computer-aided detection; *GC*, gastric cancer; *SD*, standard deviation; *H.pylori*, Helicobacter pylori: *SMD*, standardized mean difference.

∗ Atrophic border: Classified by Kimura-Takemoto classification. Closed type indicates cases where atrophy is confined to the lesser curvature, while open type indicates cases where atrophy extends to the anterior and posterior walls.

### Detection box appearance frequency

Overall, 801 detection boxes were generated under WLI, with 5 histopathologically confirmed true cancers yielding a positive predictive value (PPV) of 0.62%. Under LCI, 204 detection boxes were generated, with 5 true positives, resulting in a PPV of 2.45%. No known cancers were missed by CADe in either mode, corresponding to a sensitivity of 100%.

**Figure 3:**
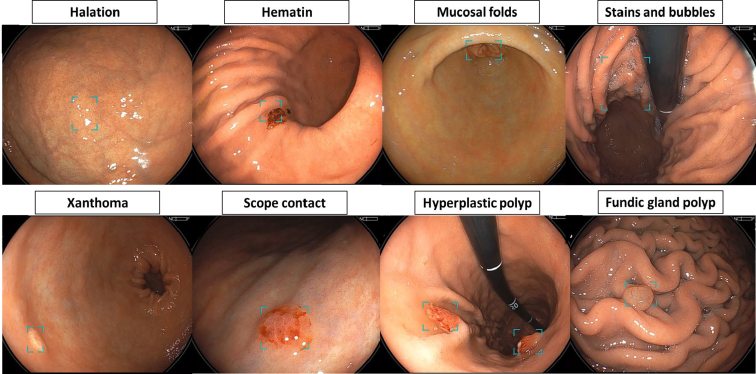
Examples of false-positive sites (*blue boxes*) using computer-aided detection. These included halation, hematin, mucosal folds, stains, bubbles, xanthomas, endoscopic contacts, hyperplastic polyps, and fundus gland polyps. Halation refers to the intense light reflection or glare on the mucosal surface.

**Figure 4:**
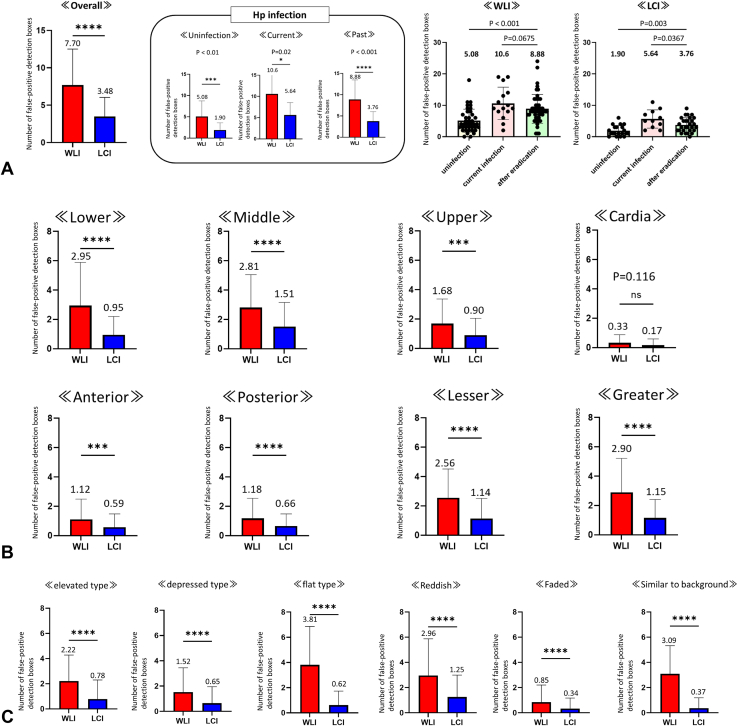
Comparison of the appearance of the false-positive detection box with that of white-light imaging (WLI) and linked-color imaging (LCI). **A,** Detection box appearance frequency in all the cases. WLI had a mean (standard deviation) of 7.70 (4.81) detection boxes, whereas LCI had an average of 3.48 (2.55), with LCI demonstrating significantly lower false-positive rates (*P* < .001) (far-left panel). Both LCI and WLI exhibited higher false-positive rates in current and past *Helicobacter pylori* infection cases than in the uninfected cases (2 far-right panels). For WLI observations, the mean number of false positives was 5.08 (3.67) in non–*H pylori* infected cases, 10.60 (5.17) in currently infected cases, and 8.88 (4.59) in previously infected cases. In contrast, LCI showed mean false-positive counts of 1.90 (1.68) in non–*H pylori* infected cases, 5.64 (2.91) in currently infected cases, and 3.76 (2.26) in past infected cases. **B,** Gastric location-specific false-positive detection box appearance frequency. LCI misidentified less at all sites except cardia. **C,** False-positive detection box appearance frequency based on the morphologic type of the lesion or surface color. LCI had lower false positives, regardless of the morphologic type or surface coloration. ∗∗∗*P* < .001, ∗∗∗∗*P* < .0001. *Hp*, Helicobacter pylori; *ns*, not significant.

Excluding sites with early-stage GCs, an average of 6.16 (4.60) detection boxes appeared in nontumor sites in each case. False-positive sites included halation (glare on the mucosal surface), hematin, mucosal folds, stains, bubbles, xanthomas, endoscopic contacts, hyperplastic polyps, and fundic gland polyps (Fig. 3). We compared the appearance of the false-positive detection box with that of WLI and LCI (Fig. 4). Overall, the average number of false-positive detection boxes was 7.70 (4.81) and 3.48 (2.55) for WLI and LCI, respectively. Paired *t* test analysis demonstrated that the number of false-positive detection boxes was significantly lower with LCI than with WLI (mean difference: 4.32; 95% CI, 5.01 to −3.64; *P* < .001). These findings suggest that CADe markedly reduces false-positive detections when used with LCI. In the state of *H pylori* infection, both LCI and WLI showed significantly higher false-positive rates for current and past infection cases than for uninfected cases. In the WLI observations, the mean (standard deviation) number of false positives was 5.1 (3.7), 10.6 (5.2), and 8.9 (4.6) in non–*H pylori* infected cases, currently infected cases, and previously infected cases, respectively, whereas in LCI, the same numbers were 1.9 (1.7), 5.6 (2.9), and 3.8 (2.3), respectively. This indicated that the LCI had a significantly lower false-positive rate in all 3 groups. Notably, according to gastric location, morphologic type, and surface color of the recognized sites, the false-positive rate was significantly lower for LCI than for WLI.

**Figure 5:**
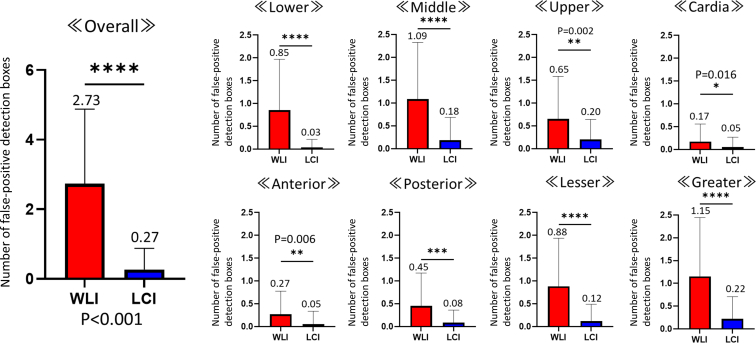
False-positive findings included halation, hematin, mucosal folds, and stains and bubbles. *LCI*, Linked-color imaging; *WLI*, white-light imaging. Significantly fewer false-positive detection boxes were seen with LCI than with WLI and across all stomach locations. ∗*P* < .05, ∗∗*P* < .01, ∗∗∗*P* < .001, ∗∗∗∗*P* < .0001.

Figure 5 shows the false-positive rates for only 4 patterns, excluding mucosal changes: halation, hematin, mucosal folds, and stains and bubbles. Interestingly, false positives unrelated to mucosal changes were significantly more frequent with WLI than with LCI, and were nearly absent in the latter, regardless of the site.

### Results of the endoscopic procedure

The mean observation time tended to be slightly longer in the CADe group than in the control group (12.1 [5.3] vs 11.1 [5.1] minutes); however, the difference was not significant (Table 2). The biopsy rates in the CADe group were slightly higher than those in the control group (22.9% vs 16.2%); however, the difference was not significant. Of all 105 CADe group patients, 7 GCs were detected in 5 patients (4.8%). In the 600-patient control group, 11 GCs were detected in 11 (1.8%) patients. The GC detection rate in the CADe group was slightly higher than in the control group (difference, 3.0%; 95% CI, 0.3%-6.3%), although the difference was not statistically significant (*P* = .07). After matching, 1 GC was detected among 105 patients in the control group (0.95%), with an absolute difference of 3.85% (95% CI, 0.67%-8.29%; *P* = .098). The mean lesion size was 9.6 (5.4) mm. All cases were histopathologically well-differentiated adenocarcinoma and located within the mucosal layer. Approximately three-quarters of the lesions were located in the gastric body, with 57.1% appearing reddish and 71.4% exhibiting surface-elevated morphology.

**Table 2:** Results of the endoscopic procedure

Comparison between CADe and conventional endoscopy					
Value	CADe group (n = 105)	Control group before matching (n = 600)	*P* value	Control group after matching (n = 105)	*P* value
Observation time, minutes, mean (SD)	12.1 (5.3)	11.3 (5.1)	.32	11.1 (5.5)	.15
Biopsy, n (%)	24 (22.9)	132 (22.0)	.89	17 (16.2)	.29
Detection of gastric cancer, n (%)	5 (4.76)	11 (1.83)	.07	1 (0.95)	.09

Newly detected early gastric cancer					
Value	CADe group, n = 5, 7 lesions	Control group (before matching), n = 11, 11 lesions	*P* value	Control group (after matching), n = 1, 1 lesion	*P* value
Size, mm, mean (SD)	9.6 (5.4)	7.9 (4.1)	.57	10.0 (0.0)	1.00
Histopathology tub1, n (%)	7 (100)	11 (100)	1.00	1 (100)	1.00
Depth: mucosa, n (%)	7 (100)	11 (100)	1.00	1 (100)	1.00
Location: lower/middle/upper, n (%)	1 (14.3) / 5 (71.4) / 1 (14.3)	3 (27.3) / 7 (63.6) / 1 (9.1)	.79	1 (100.0) / 0 (0.0) / 0 (0.0)	.18
Color: reddish/similar to background, n (%)	4 (57.1) / 3 (42.9)	8 (72.7) / 3 (42.9)	.62	0 (0.0) / 1 (100.0)	.28
Morphologic type: 0-Ila/0-Ilb/0-Ilc, n (%)	4 (57.1) / 0 (0.0) / 3 (42.9)	5 (45.5) / 2 (18.2) / 4 (36.4)	.48	0 (0.0) / 2 (18.2) / 4 (36.4)	.01

*0-Ila*, Flat and elevated, *0-Ilb*, completely flat; *0-Ilc*, superficially depressed; *CADe*, computer-aided detection; *SD*, standard deviation; *tub1*, well-differentiated tubular adenocarcinoma.

### Recognition level of the landmark checker

With the exception of 1 patient who underwent surgery for GC, landmark checker site recognition was investigated retrospectively in 104 patients. All gastric sites indicated by the landmark checker were observed in all patients. Conversely, an average of 5.72 of 7 sites (81.7%) were recognized by CADe (Table 3). The middle of the lesser curvature and the body of the greater curvature were identified by CADe in nearly all cases (98.1% and 90.4%, respectively), whereas the angle of the lesser curvature was not recognized in more than half of the cases (53.8%). There was no significant difference based on whether the endoscopist was board-certified.

**Table 3:** Recognition level of the landmark checker

Value	Location (n = 104) (1 patient after gastrectomy was excluded)							
	Overall (mean)	EGJ	Antrum	Angle of lesser curvature	Middle of lesser curvature	Upper of lesser curvature	Cardia	Body of greater curvature
All endoscopists, (n = 104)	5.72	87	89	48	102	91	84	94
%	81.7	83.7	85.6	46.2	98.1	87.5	80.8	90.4
Board-certified, (n = 84)	5.77	68	74	41	82	74	70	76
%	82.5	81	88.1	48.8	97.6	88.1	83.3	92.5
Young resident, (n = 20)	5.5	19	15	7	20	17	14	18
%	78.6	95	75	35	100	85	70	90
*P* value	.27	.18	.15	.32	1.00	.71	.20	1.00

*EGJ*, Esophagogastric junction.

### Diagnosis of known early GC

Eighteen patients with known early GC during the study period were examined to determine whether CADe could detect the lesions (Table 4). The mean patient age was 77.5 years (9.7), and 88.9% were male. The mean size of the lesion was 14.7 mm (11.7). All the lesions were located within the mucosal and submucosal layers. About one-third of the lesions were surface-depressed (38.9%), and one-third were surface-elevated (38.9%). CADe accurately identified all lesions.

**Table 4:** Characteristics of known early gastric cancer all detected by CADe (n = 18)

Characteristic	Value
Age, years, mean (standard deviation)	77.5 (9.7)
Sex: men/women, n (%)	16 (88.9) / 2 (11.1)
Size, mm, mean (standard deviation)	14.7 (11.7)
Location: lower/middle/upper part, n (%)	3 (16.7) / 14 (77.8) / 1 (5.6)
Histopathology: tub1/tub2/por or sig, n (%)	14 (77.8) / 1 (5.6) / 3 (16.7)
Depth: mucosa/submucosa, n (%)	14 (77.8) / 4 (22.2)
Morphologic type: 0-IIa/0-IIb/0-IIc/0-I, n (%)	7 (38.9) / 3 (16.7) / 7 (38.9) / 1 (5.6)

*0-I*, Polypoid; *0-IIa*, flat and elevated; *0-IIb*, completely flat; *0-IIc*, superficially depressed; *CADe*, computer-aided detection; *por*, poorly differentiated tubular adenocarcinoma; *sig*, signet ring cell carcinoma; *tub1*, well-differentiated tubular adenocarcinoma; *tub2*, moderately differentiated tubular adenocarcinoma.

## Discussion

To our knowledge, this study is the first to evaluate the real-world performance of a newly developed CADe, CAD EYE, in detecting GC during upper GI endoscopic examinations. The results demonstrated that CADe for the upper GI tract still has a high false-positive rate, and LCI may help improve PPV. Furthermore, CADe could detect unknown GC cases in the routine setting and all known upper GI cancers, although there were no significant differences in comparison with the control group.

Various developments have been made to improve the diagnostic performance of EGD for GC and gastritis, including image-enhanced endoscopy (IEE). In addition to the classical indigo carmine dye method, a newly developed IEE using optical digital methods such as LCI and third-generation narrow-band imaging has been introduced recently worldwide.^18^, ^19^, ^20^ Furthermore, the development of AI-based endoscopic diagnostic aids has progressed rapidly in recent years.

CE was the first CADe for the upper GI tract to be approved by the Japanese Pharmaceuticals and Medical Devices Agency. In this study, the GC detection rate was higher in the CADe group than in the control group, although the difference was not statistically significant. Notably, multiple simultaneous GCs were found in the CADe group, whereas no simultaneous cancers were present in the control group. Endoscopists sometimes become preoccupied with finding a single lesion and consequently overlook simultaneous cancers; however, this may be prevented by using CADe in combination with LCI. Furthermore, the use of CADe did not increase the overall examination time or biopsy rate compared with conventional EGD. Although the primary outcome focused on the intragroup comparison of false-positive detection rates between WLI and LCI modes within CADe-assisted procedures, the inclusion of a control group allowed for secondary outcome analyses. These comprised assessing whether the adoption of CADe affected key clinical parameters, such as biopsy rates or examination time, which are important considerations for real-world applicability.

Our results indicated that CADe can be integrated into routine practice without substantially prolonging the procedure or increasing unnecessary biopsies. Furthermore, CADe accurately detected all known early upper GI cancers examined during the study period, demonstrating high sensitivity for detecting established lesions. Conversely, CADe exhibited a relatively high false-positive rate, with an average of 6.2 detection boxes appearing in nontumor sites per case. This finding is likely due to the fact that the gastric mucosa is modified by *H pylori* infection, which causes several mucosal changes similar to those of GC. Furthermore, neoplastic lesions in the stomach are known to shrink, flatten, or obscure after *H pylori* eradication, making it difficult to endoscopically diagnose whether the lesion is a GC or gastritis.^21^, ^22^, ^23^ Furthermore, our study indicated that the false-positive rates of CADe in patients with current or past *H pylori* infection were significantly higher than those in the uninfected patients. Notably, the false-positive rate was significantly lower when using LCI than when performing WLI. Regardless of the part of the gastric body, morphologic type, or surface color of the lesion, LCI was consistently superior to WLI, and there were almost no false positives in the LCI for lesions other than mucosal changes, such as bubbles or dirt. A combination of LCI has been reported to be beneficial for AI diagnosis of *H pylori* infection.^24^ However, there have been no reports on the combination of CADe and IEE for the detection of GCs. Our results suggest that LCI helps improve the specificity of CADe. Regarding landmark recognition, the landmark checker function of CADe successfully recognized an average of 5.72 out of 7 key gastric sites (81.7%). Nonetheless, the recognition rates varied by location, with the angle of the lesser curvature being the most challenging site to identify. Overall, the recognition rate was relatively high and was not inferior to the site classification diagnosis rate (65.9%) of the existing AI reported by Wu et al.^25^

Beyond diagnostic accuracy, false-positive detections can have important resource implications, including unnecessary biopsies, prolonged procedure times, increased pathology workload, and potential patient anxiety. Although our study did not observe a significant increase in biopsy rates, the presence of numerous nonneoplastic detection boxes—particularly under WLI—may contribute to downstream inefficiencies. Lessons from colonoscopy-based CADe systems suggest that optimizing the balance between sensitivity and specificity is critical not only for clinical accuracy but also for workflow efficiency.^7^ Incorporating strategies such as AI-assisted decision support or real-time confidence scoring, as explored in colorectal applications, may help mitigate the impact of false positives in EGD.

We observed that certain lesions, such as fundic gland polyps and xanthomas, frequently triggered false positives because of their irregular surface and coloration. These findings suggest that mucosal pattern complexity in the gastric corpus remains a challenge for the current CADe systems. Further algorithmic refinement and enhanced feature training may be needed to differentiate these entities more reliably.

This study has some limitations. First, it was conducted at a single center, which may limit generalizability. Second, the number of newly detected GCs was small, limiting the statistical power to compare detection rates. Larger studies are needed to assess the impact of CADe on GC detection rates and clinical outcomes more definitively. Third, in evaluating the false-positive rate, most of the CADe-detected regions were not biopsied. Although pathologic confirmation is the reference standard for cancer diagnosis, it is neither practical nor ethically appropriate to perform biopsies on all AI-indicated regions, particularly when many are clearly benign upon endoscopic visual inspection. Therefore, our definition of false positives relied on the endoscopists' diagnoses and expert video review. Although this approach may introduce some subjectivity, it reflects real-world clinical practice and allows for practical assessment of CADe system performance in a routine endoscopy setting. Fourth, the observation method for the control group was not specified, making it unclear whether IEE was used in the control group.

In conclusion, this study provides initial evidence that CADe, particularly when combined with LCI, has the potential to enhance GC detection during routine EGD without prolonging examination time. Although promising, further optimization to improve specificity and larger validation studies are needed.

## Data availability statement

The data sets used and analyzed in the current study can be acquired from the corresponding author upon reasonable request.

## Statement of ethics

The study was conducted in accordance with the Declaration of Helsinki of the World Medical Association and the Ethical Guidelines for Medical and Health Research Involving Human Subjects established by the Ministry of Health, Labour, and Welfare, Japan.

## Study approval statement

The study protocol was reviewed and approved by Akashi City Hospital (Hyogo, Japan) (approval number: 2024005).

## PATIENT CONSENT

The requirement for written informed consent from the patients was waived due to the retrospective nature of the study. Therefore, an opt-out method was used to collect clinical data for this study.

## DISCLOSURE

All authors disclosed no financial relationships.
